# Personalized dental medicine, artificial intelligence, and their relevance for dentomaxillofacial imaging

**DOI:** 10.1259/dmfr.20220335

**Published:** 2022-12-12

**Authors:** Kuo Feng Hung, Andy Wai Kan Yeung, Michael M. Bornstein, Falk Schwendicke

**Affiliations:** 1 Division of Oral and Maxillofacial Surgery, Faculty of Dentistry, The University of Hong Kong, Hong Kong SAR, China; 2 Division of Oral and Maxillofacial Radiology, Applied Oral Sciences and Community Dental Care, Faculty of Dentistry, The University of Hong Kong, Hong Kong SAR, China; 3 Department of Oral Health & Medicine, University Center for Dental Medicine Basel UZB, University of Basel, Basel, Switzerland; 4 Department of Oral Diagnostics, Digital Health and Health Services Research, Charité–Universitätsmedizin Berlin, Berlin, Germany

**Keywords:** personalized medicine, artificial intelligence, deep learning, diagnostic imaging, dentistry

## Abstract

Personalized medicine refers to the tailoring of diagnostics and therapeutics to individuals based on one’s biological, social, and behavioral characteristics. While personalized dental medicine is still far from being a reality, advanced artificial intelligence (AI) technologies with improved data analytic approaches are expected to integrate diverse data from the individual, setting, and system levels, which may facilitate a deeper understanding of the interaction of these multilevel data and therefore bring us closer to more personalized, predictive, preventive, and participatory dentistry, also known as P4 dentistry. In the field of dentomaxillofacial imaging, a wide range of AI applications, including several commercially available software options, have been proposed to assist dentists in the diagnosis and treatment planning of various dentomaxillofacial diseases, with performance similar or even superior to that of specialists. Notably, the impact of these dental AI applications on treatment decision, clinical and patient-reported outcomes, and cost-effectiveness has so far been assessed sparsely. Such information should be further investigated in future studies to provide patients, providers, and healthcare organizers a clearer picture of the true usefulness of AI in daily dental practice.

## Personalized and precision dentistry and data-driven technologies

Current concepts of managing dental diseases have by large been developed over the course of the last 50 years. While knowledge generated by continuous research efforts towards the biological foundation of the main dental diseases (caries and periodontitis) has been gradually integrated into contemporary therapy approaches, the backbone of treatments employed in dental practices has been established decades ago. For example, restorative treatments remain the cornerstone for carious lesions while deep scaling and root planing remain central for periodontal disease, both of which are increasingly accompanied by preventive efforts.^1,2^ Given the evolving understanding of dental diseases, their etiology and pathogenesis, and the resulting chance and need to adequately describe different disease stages and grades to deduct appropriate therapies, this simplification may not suffice any longer. Notably, it is grounded in a similarly simplified diagnostic approach; what is missing is a systematic and holistic evaluation of individual health and disease on patient, tooth, and site level, and the synthesis of the gathered data into adequately granular diagnoses. Such an approach would need to be built on a detailed multimodal data collection and would allow to assign individualized treatment pathways based on personalized diagnosis.

At present, however, such individualized diagnostics and treatment pathways are not at all available in dentistry. Instead, we are stuck in the era of stratification of individuals and lesions into risk groups, characterized mainly by simple shared phenotypic characteristics (*e.g.,* caries experience for caries risk assessment, smoking or poor oral hygiene for periodontal risk assessment, etc.). Currently, the accuracy and generalizability of most of these risk assessment systems are insufficiently validated. Even if these risk assessment systems were valid, they would only describe groups of individuals and lesions sharing a similar “risk” and subsequently assign identical management strategies to all individuals in a certain risk group (*i.e.,* the one-size-fits-all approach).^3^

**Figure 1.**
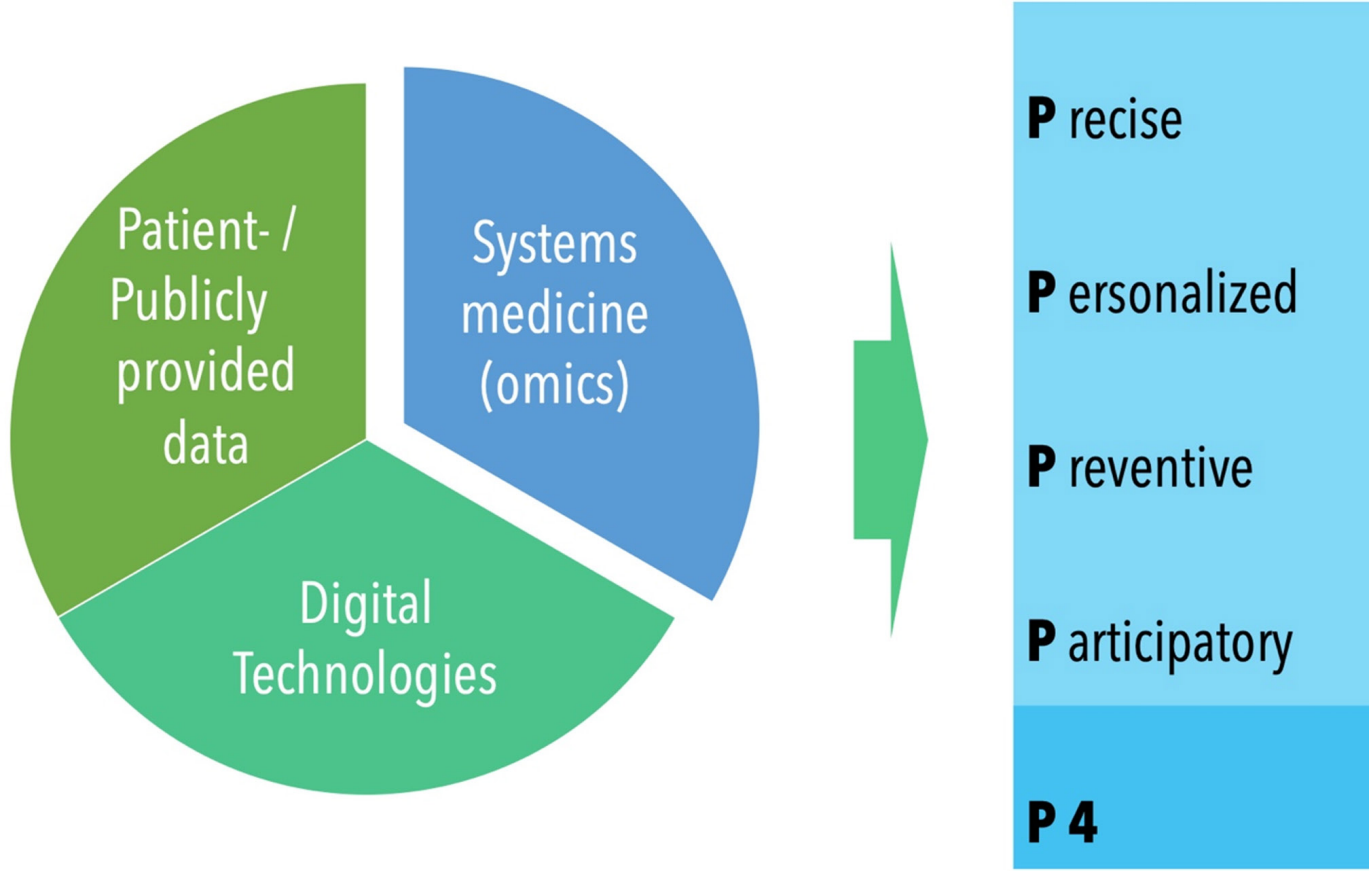
The confluence of different data sources and technologies (*e.g.,* AI, specifically deep learning, systems medicine involving genomic, metabolomic, or microbiomic data, as well as clinical data sources or those provided publicly or by the patient) will enable P4 medicine and dentistry.^4^

While being the next step beyond stratification, true personalized management is not possible at the moment. Personalized management is closely linked to “precision medicine”, defined as “the tailoring of a therapy to individuals with one’s biological (genomic, microbiomic, proteomic, etc.), social (economic, educational, etc.) and behavioral (lifestyle) characteristics”.^3^ Personalized care should, ideally, allow to provide the safest, most efficacious and efficient diagnostics and therapies, which is jointly with precision medicine and closely related to another concept “P4 medicine”.^4^ The four Ps stand for a more precise, personalized, preventive, and participatory healthcare approach (Figure 1). What is needed, however, to make personalized, precision, and P4 dentistry come true is a deep understanding of individuals and the option to predict what will happen to this individual, a specific organ or lesion.

To allow such understanding and prediction, the discussed concept of stratification and the employed few risk indicators or factors (Table 1) are obviously insufficient. What is needed is a shift towards a healthcare model centered around broad and deep data. As discussed elsewhere,^7^ many recent academic breakthroughs in astronomy,^8^ biology^9^ and other disciplines are mainly driven by making use of large amounts of data. Dentistry should also make use of the wealth of available dental data and transform into something that was previously referred to as “data dentistry”.^7^ The data needed could be generated from advanced sensor technologies, including wearables, ingestibles, and implantables as well as social media and electronic health records (eHR), to name a few.^10^ Many of these data sources will not solely rely on being collected in clinical settings, but routinely, even by patients who may actively donate data from social media, food consumption, healthcare apps, behavioral diaries, or toothbrushes. In addition, prospectively collected omics data may become more available if costs for generating them decrease further and technologies are becoming available in routine settings.^10^

**Table 1.** Risk factors and risk indicators

	Risk factor	Risk indicator
**Definition**	“A characteristic that may make an individual more susceptible to a certain disease”^3^ ; can be “environmental, behavioral, or biologic and “if present directly increases the probability of a disease occurring, and if absent or removed reduces the probability”.^5^	“A marker that is not necessarily causally linked, but can be used to predict risk, like past disease experience or social, educational or economic factors” ^3^; “may be a probable, or putative, risk factor, but […] a temporal association usually cannot be specified”.^6^
**Caries**	Diet rich in carbohydrates	Caries experience
	Oral hygiene, fluoridated toothpaste	Low social, educational or economic status
	Medication causing hyposalivation/xerostomia	
**Periodontal disease**	Oral hygiene, smoking	Periodontitis experience
	Bacterial composition, genetic factors (SNPs)	Low social, educational or economic status
	Medication inducing immunosuppression	
**Oral cancer**	Smoking	Low social, educational or economic status
	Betel quid	Male sex
	Alcohol consumption	High age

## Artificial intelligence and its use in dental medicine

The analysis of such diverse, multimodal, large, and complex data, including speech and imagery, requires advanced data analytic approaches.^11^ One major strategy adopted over recent years for this purpose is “artificial intelligence” (AI). The term was coined in the 1950’s and refers to the idea of building machines that are capable of performing tasks that are normally performed by humans. Machine learning (ML) is a subfield of AI where algorithms are applied to learn the inherent statistical patterns and structures in data, which allows for predictions of unseen data. More complex machine learning algorithms frequently used for data like images are neural networks (NNs), which are constituted of artificial neurons (*i.e.,* mathematical non-linear models that can be stacked and concatenated in layers using mathematical operations to form a network). The term “deep learning” is a reference to deep (multilayered) NNs, which are able to represent hierarchical features in complex data and frequently used for detecting edges, corners, shapes, and macroscopic patterns in images.^12^

ML and NNs as a subtype of AI are “trained” to automatically perform specific tasks, and the most common type of training is supervised learning where data points and corresponding data information (*e.g.,* labels, tasks, etc.) are repetitively passed through the network to detect the intrinsic statistical patterns in the data. During the training process, the connections between the neurons, also referred to as model weights, are optimized to minimize the so-called prediction error (difference of the true *vs* the predicted data information). A trained NN can predict the outcome of unseen data by passing the new data point through the network. AI, ML, and NNs are increasingly used in dentistry to work with the increasing amount of data available, as described above. A number of such forms of use are currently discussed or already clinically available:

### 1. Data analytics and precision dentistry

As discussed, there is an increasing strive towards more precise data-centered dentistry, making use of not only clinical and historical data, claims and treatment data, image and further test data, but also data provided by patients as outlined above. A big advantage in dentistry is that these multimodal datasets are usually available repeatedly as many patients visit dentists regularly. Using such longitudinal data will help to foster a deeper understanding of individual health and disease and to develop AI models to predict disease onset or progression individually. Currently, however, many of these data remain siloed or unavailable. Meanwhile, existing AI prediction models remain limited in their predictive power and generalizability as useful predictions need to be better than plainly guessing the so-called majority class (*i.e.,* the more frequent event).^3^ Predicting this majority class is easy but models which focus on exclusively predicting it may not be clinically useful.^13^

### 2. Evidence-based care

Gathering a more comprehensive picture of an individuals’ health and objectifying diagnosis through imagery and AI-assisted analysis will support evidence-based care. Data-centric approaches will further allow to embed external evidence, for example from guidelines and standards of care, into decision making, and then fostering reliable high-quality and cost-effective care.^14^ An additional benefit of more data-driven care is the option to objectively assess treatment needs, actually provided treatments, and the yielded outcomes. Ultimately, this should foster value-based care (*i.e.,* quantifying the “value” of a certain treatment to individuals and the society).

### 3. Beyond the dental chair

AI and data-driven approaches will facilitate better information and decision making on the dental public health level, including workforce planning. Automated data generation in routine settings in addition to prospective epidemiologic surveys will allow a more up-to-date and detailed picture of a populations’ health, its oral health demands, and the effectiveness and efficiency of services.^15^ This will facilitate the informed setup or buy-in of services as well as benchmarking of healthcare interventions and policy. It will support a needs-based and adaptive workforce planning. Enabling providers of different levels, AI and data-driven approaches will further support modularized models of care, fostering affordable, accessible, and specialized services. AI will further change dental education by employing non-synchronous learning models. Learning using simulation including augmented or virtual reality-based teaching and training will be more common in the future.^16^

## Current use of AI in dentomaxillofacial imaging

Radiographic examination is an integral component in most diagnostic and treatment planning processes in daily dental practice. With the growing use of digital dental radiography, images generated by dental radiographic examinations are commonly automatically stored as digital data in the archiving system and associated databases. These data can be analyzed using AI and specifically deep learning based on convolutional NNs.^17^ Currently, a range of deep learning models have been trained and tested on dentomaxillofacial radiographic images to fulfill tasks of image classification (*e.g.,* “is there a certain pathology detectable on this image?”), object detection (*e.g.,* “in which image area is this certain pathology located?”) and pixelwise segmentation (*e.g.,* “which pixels of this image show a certain pathology”), and for image quality improvement (Table 2).^95,96^

**Table 2.** Artificial intelligence applications using dentomaxillofacial imaging data

Category	Artificial intelligence application
Dental caries	Detection of dental caries^18–21^
Periodontal evaluation	Detection of periodontal bone loss^22,23^
	Measurement and staging of periodontal bone loss^24,25^
	Classification of periodontitis stages^26,27^
	Identification of periodontally compromised teeth^28,29^
Endodontic evaluation	Detection, classification, and measurement of apical pathologies^30,31^
	Detection of vertical root fractures^32,33^
	Detection^34^ and classification^35,36^ of C-shaped canals
Dental implants	Detection of peri-implant bone loss^37^
	Measurement of the peri-implant bone loss ratio and classification of the bone loss severity^38^
	Detection of the edentulous sites, nasal fossa, maxillary sinus, and mandibular canal, and measurement of the heights and widths of residual alveolar bone at the edentulous sites^39^
	Classification of dental implant systems^40–42^
	Detection and classification of dental implant fractures^43^
Third molars	Classification of positional relationships between lower third molars and the mandibular canal^44–46^
	Prediction of extraction difficulty for lower third molars^47^
	Prediction of paresthesia after third molar extraction^48^
Radiolucent lesions in the jaws	Detection and segmentation of infections, granuloma, cysts, and tumors in the jaws^49^
	Detection of ameloblastomas and odontogenic keratocysts^50^
	Detection/classification of ameloblastomas, odontogenic keratocysts, dentigerous cysts, radicular cysts, and/or bone cysts in the maxilla/mandible^51,52^
	Differentiation of Stafne’s bone cavity from mandibular radiolucent lesions^53^
Maxillary sinus	Detection of maxillary sinus lesions^54,55^
	Detection and segmentation of maxillary sinus lesions^56,57^
	Prediction of oroantral communication after tooth extraction^58^
Orthodontic and orthognathic evaluation	Localization of cephalometric landmarks^59–64^
	Classification of skeletal malocclusion^65–67^
	Assessment of facial symmetry before and after orthognathic surgery^68^
Temporomandibular joint	Diagnosis of temporomandibular joint osteoarthritis^69^
	Diagnosis of mandibular condyle fractures^70^
	Measurement of the cortical thickness of mandibular condyle head^71^
Maxillofacial fracture	Detection and classification of mandibular fracture^72^
Sialoliths	Detection of submandibular gland sialoliths^73^
Osteoporosis	Diagnosis and prediction of osteoporosis^74,75^
Sjögren’s syndrome	Diagnosis of Sjögren’s syndrome^76^
Lymph node metastasis	Segmentation and identification of metastatic cervical lymph nodes^77^
Reporting of the dental status	Segmentation of teeth and jaws, numbering of teeth, detection of caries, periapical lesions, and periodontitis^78^
	Identification of missing tooth, caries, filling, prosthetic restoration, endodontically treated tooth, residual root, periapical lesion, and periodontal bone loss^79^
	Tooth numbering and detection of dental implants, prosthetic crowns, fillings, root remnants, and root canal treatment^80^
	Detection, segmention, and labeling of teeth, crowns, fillings, root canal fillings, implants, and root remnants^81,82^
	Tooth detection and numbering^83,84^
	Tooth segmentation and classification^85,86^
Image quality improvement	Correction of blurred panoramic radiographic images^87^
	Reduction of metal artifacts on CBCT images^88^
	Improvement of the resolution of CT/CBCT images^89–91^
Multimodal image registration	Registration of CBCT with intra oral scan,^92^ optical dental model scan,^93^ or MRI^94^

## Diagnosis

### 1. Dental caries

Intraoral radiographic examination is essential for the detection of dental carious lesions, particularly early non-cavitated ones. The sensitivity and specificity of intraoral radiography for detecting dental caries were reported to range from 27–66% and 76–97%, respectively.^97,98^ The relatively low sensitivity reported implies a high underdetection of dental caries, which may be related to clinicians’ experience and caries lesion depth (*i.e.,* enamel or dentin caries).

Several deep learning models have been developed to assist clinicians in detecting and classifying dental caries.^96^ Lee et al developed three CNN models to automatically detect dental caries in posterior teeth on periapical images.^18^ The models showed higher detection accuracy for premolars than for molars, which could be related to differences in their anatomical characteristics. Srivastava et al developed a CNN model to detect dental caries on bitewings. The AI model achieved significantly higher sensitivity (81%) than three general dentists (34–48%).^19^ More recently, caries detection using AI additionally focused on the detection of early enamel caries. The CNN model developed by Cantu et al outperformed seven experienced dentists in detecting initial enamel and advanced dentin caries.^20^ The seven dentists showed greatly different sensitivities for detecting initial (<25%) and advanced (40–75%) caries while the model achieved robust sensitivities (>70%) for both initial and advanced caries. Currently, commercial AI software programs including AssistDent (Manchester, UK), Denti.AI (Toronto, Canada), Diagnocat (Tel Aviv, Israel), CranioCatch (Eskişehir, Turkey) and dentalXr.ai (Berlin, Germany) (Table 3) are available to assist clinicians in the diagnosis of dental caries on two-dimensional (2D) radiographic images. The use of AssistDent and dentalXr.ai significantly increased dentists’ sensitivity especially for detecting enamel caries.^21,99^ Notably, automatic detection of buccal/lingual caries or secondary caries (*i.e.,* caries next to restorations) remains challenging for AI models. This is, however, also the case for human observers and mainly grounded in the 2D nature of most intraoral images. While cone-beam computed tomography (CBCT) allows caries detection in three-dimensions (3D), it is not recommended for caries diagnostics.

**Table 3.** Examples of commercially available AI software for dental applications

AI software	Origin (City, Country)	Type of image data	Application	Website
AssistDent	Manchester, UK	Bitewing images	Detecting proximal enamel and dentin caries	https://www.assistdent.net
WebCeph	Seongnam, Korea	Cephalometric images	Cephalometric tracing and analysis	https://webceph.com/en/about/
Ceppro	Seoul, Korea	Cephalometric images	Cephalometric tracing and analysis	https://www.ddhinc.net/en/
dentalXr.ai	Berlin, Germany	Bitewing, periapical and panoramic images	Identifying and numbering teeth Detecting caries, apical lesions, fillings, crowns, bridges, dental implants, root canal fillings, retained teeth, calculus, and periodontal bone loss Anatomical structure segmentation Generating findings report	https://www.dentalxr.ai/en/home
Relu	Leuven, Belgium	CBCT images	Identifying and numbering teeth Segmenting teeth, jaws, mandibular canal, and pharyngeal airway	https://relu.eu/
Denti.AI	Toronto, Canada	Periapical, bitewing, panoramic, and CBCT images	Identifying and numbering teeth Detecting caries, fillings, apical lesions, and endodontic treatment Charting for dental X-rays, CBCT, and voice data	https://www.denti.ai/
Promaton	Amsterdam, Netherlands	Panoramic and CBCT images	Identifying and numbering teeth Detection of dental implants, prosthetic crowns, fillings, root remnants, and root canal treatment Tooth segmentation Dental implant planning Alignment of optical and CBCT scans	https://www.promaton.com
Diagnocat	Tel Aviv, Israel	Periapical, bitewing, panoramic, cephalometric, and CBCT images	Identifying and numbering teeth Detecting caries, apical lesions, periodontal bone loss, open margins, overhangs, impactions, filling, prosthetic restoration, endodontically treated tooth, calculus, and residual root. Anatomical structure segmentation Generating findings report	https://diagnocat.com
CranioCatch	Eskişehir, Turkey	Periapical, bitewing, panoramic, cephalometric, and CBCT images	Identifying and numbering teeth Detecting caries, apical lesions, impacted teeth, alveolar bone loss, furcation defects, jaw pathologies, and dental restorations Evaluating bone changes in the temporomandibular joint Orthodontic analysis Anatomical structure segmentation Treatment plan recommendation	https://www.craniocatch.com/en/

AI, artificial intelligence; CBCT, cone-beam computed tomography

### 2. Periodontal bone loss

Deep learning models have also been developed for the detection and segmentation of periodontal bone loss and the associated classification of periodontitis stages on periapical and panoramic images. In 2018, Lee et al developed a CNN model on periapical images to automatically identify periodontally compromised posterior teeth and predict tooth loss in the future.^28^ The accuracy of the model was higher for premolars (>80%) than for molars. Thanathornwong et al developed a CNN model to identify periodontally compromised teeth on panoramic images.^29^ Kim et al^22^ and Krois et al^23^ trained their CNN models to automatically detect periodontal bone loss on panoramic radiographs. The diagnostic accuracies of their models (AUCs [area under the curves] of 0.89–0.95) were higher than that of several general dentists (AUCs of 0.77–0.85).

In addition, periodontitis stages can also be classified automatically using deep learning on periapical and panoramic images.^26,27^ Danks et al^24^ and Lee et al^25^ developed CNN models to measure the extent of periodontal bone loss on periapical images and subsequently classify the identified sites into three/four severity stages according to the bone loss extent measured. The model by Lee et al achieved high classification accuracy with an AUC value of 0.98. Future applications are expected to detect changes in the bone density and textures of the alveolar ridge for early detection of the onset of periodontitis.

### 3. Endodontic evaluation

AI applications in endodontics so far mainly focus on apical pathologies, root fractures, and C-shaped canals. For apical pathologies, models on 2D radiographic images were able to automatically detect and classify lesions while those on CBCT images were able to additionally provide volumetric information of the detected lesions. Krois et al^100^ and Ekert et al^30^ developed CNN models on panoramic images or image crops to detect apical pathologies and classify teeth into (i) teeth without apical lesions, (ii) teeth with widened periodontal ligament or uncertain apical lesions, and (iii) teeth with clearly visible apical lesions. Krois et al trained their CNN models with images acquired from one or two centers, respectively, and reported low cross-center generalizability of the model trained with images acquired only from one center.^100^ The low generalizability mainly resulted from differences in the dental status shown on images from different centers, specifically the association between root canal fillings and apical lesions being present differed. CNNs learnt this association structure on data from one center (where root canal fillings were frequent) but were then unable to reproduce this based on data from the other center (where root canal fillings were less frequent). Based on their findings, cross-center training seems to be able to improve a model’s generalizability. Hamdan et al reported that the diagnostic ability of eight dental practitioners to detect apical radiolucencies on periapical images increased with the aid of a commercially available AI software named Denti.AI (Toronto, Canada; Table 3), as demonstrated by an increased sensitivity from 59.6 to 73.3%.^101^ Orhan et al used 109 CBCT scans to test an AI software named Diagnocat (Tel Aviv, Israel; Table 3) and reported high detection accuracy and no significant differences in the lesion volumes measured by the software and a radiologist.^31^ Notably, the presence of endo-perio lesions, buccal-lingual cortical perforations, incomplete apex, endodontically treated teeth, and large lesions associated with multiple teeth detrimentally affected the model’s performance.

Diagnosis of root fractures, especially vertical root fractures, is a challenging and experience-dependent task, commonly accomplished by combined clinical and radiographic examination. Root fractures are categorized as horizontal and vertical fractures. Horizontal root fractures frequently occur in the anterior teeth due to dentoalveolar trauma while vertical root fractures are common in endodontically treated teeth as a result of excessive root canal preparation or occlusive force. CNN models have been developed to automatically detect vertical root fractures on 2D and 3D radiographic images.^32,33^ Despite promising diagnostic accuracy, the models still have to overcome a relatively low accuracy on non-endodontically treated teeth and the potential impact of caries, fillings, dental restorations, and metal artifacts on their performance.

Automatic detection and classification of C-shaped canals in mandibular second molars have been seen as another field of AI application. Several CNN models on 2D and 3D radiographic images have been developed to automatically detect, segment, and classify C-shaped canals.^34–36^ Their performance has been shown similar or superior to both general dentists and specialists.^34,36^

### 4. Dental implants

CNN models were also developed to detect peri-implant bone loss and implant fractures on 2D radiographic images. Liu et al developed a CNN model to automatically detect peri-implant bone loss on periapical images.^37^ The model performed similarly to two general dentists but inferior to one specialist. Another CNN model on periapical images measured peri-implant bone loss ratio and classified the bone loss severity into normal, early, moderate, and severe.^38^ Lee et al developed CNN models on periapical, panoramic, or both images to detect implant fractures and to classify the fractured implants into horizontal or vertical fractures.^43^ The models achieved AUCs of 0.90–0.98 for the detection task and 0.75–0.87 for the classification task. The highest detection and classification accuracies were achieved on periapical images, likely due to higher spatial resolution of periapical images compared with panoramic images.

### 5. Maxillofacial pathologies

Treatment options and prognosis for patients with pathologies in the maxillofacial region are directly associated with the timing and accuracy of diagnosis. The differential diagnosis of maxillofacial pathologies is a challenge for general practitioners, particularly for incidental findings on diagnostic images. A delayed diagnosis will lead to a longer disease course, more invasive surgical approach, and poorer treatment outcome, especially for malignant lesions. Several researchers tried to develop AI tools to improve the diagnostic accuracy of general practitioners for various maxillofacial pathologies to reach the level of specialists. Poedjiastoeti et al developed a CNN model on panoramic images for automatic detection of ameloblastomas and odontogenic keratocysts, with high diagnostic performance (sensitivity and specificity over 80%) being on par with five oral-maxillofacial surgeons.^50^ Another CNN model on panoramic images detected and classified ameloblastomas, odontogenic keratocysts, dentigerous cysts, and radicular cysts, and obtained high classification performance with an AUC of 0.94, sensitivity of 88.9%, and specificity of 97.2%, respectively.^51^ The model by Endres et al outperformed 14 oral-maxillofacial surgeons in detecting infections, granuloma, cysts, and tumors in the jaws on panoramic images.^49^ Lee et al developed CNN models, respectively, on panoramic and CBCT images to detect, segment, and classify odontogenic keratocysts, dentigerous cysts, and radicular cysts.^52^ The model on CBCT images (AUC = 0.91) outperformed the one on panoramic images (AUC = 0.85). Ariji et al et al developed a CNN model on contrast-enhanced CT images to identify and segment metastatic cervical lymph nodes in patients with oral cancer.^77^ The model outperformed two radiologists in identifying cervical lymph nodes while its segmentation accuracy should be improved.

It has been reported that inexperienced oral-maxillofacial radiologists are prone to miss pathological changes of the parotid gland while interpreting CT images of the maxillofacial region, leading to underdetection of Sjögren’s syndrome.^76^ Kise et al developed a CNN model to assess the texture features of the parotid gland on CT images for automatic diagnosis of Sjögren’s syndrome.^76^ The model performed similarly to three experienced radiologists and superior to three inexperienced radiologists.

The maxillary sinus is the largest paranasal sinus and frequently involved in dental surgical procedures due to its close proximity to the teeth in the posterior maxilla. Accurate diagnosis of maxillary sinus pathologies is the key to the success of dental surgical procedures, such as sinus augmentation for dental implant placement and apical surgery of maxillary posterior teeth.^102–104^ However, it has been reported that inexperienced dental practitioners were less likely to accurately diagnose sinus pathologies on radiographic images.^105^ In order to assist clinicians in the diagnosis of the sinus pathologies, CNN modes have been developed to automatically detect and segment sinus lesions on panoramic and CBCT images.^54–57^ The models obtained favorable performance on both detection and segmentation tasks. Murata et al reported that their CNN model performed similarly to two radiologists and outperformed two dental residents in the diagnosis of maxillary sinusitis.^55^ The CNN model by Hung et al obtained high accuracy for detecting and segmenting mucous retention cysts and mucosal thickening of the sinus on both ultra-low-dose and standard-dose CBCT images with AUCs ranging from 0.84 to 0.93.^56^

### 6. Temporomandibular joint

Diagnosis of temporomandibular joint (TMJ) disorders requires sufficient clinical experience. Undetected TMJ problems can result in patients suffering for a long time and undergoing unnecessary examinations and even invasive treatment. Jung et al developed two CNN models on panoramic images using different pre-trained flameworks for automatic diagnosis of TMJ osteoarthritis. The models achieved excellent diagnostic accuracy superior to that of three general dentists and even three TMJ specialists.^69^ The CNN model by Kim et al obtained high accuracy for measuring cortical thickness of the mandibular condyle head on CBCT images.^71^ Nishiyama et al developed CNN models to diagnose mandibular condyle fracture on panoramic images, and reported high diagnostic accuracy with AUCs of nearly 0.9.^70^

### 7. Other diagnostic purposes

Apart from the abovementioned diagnostic purposes, deep learning models can also be developed for automatic detection and classification of mandibular fractures,^72^ diagnosis and prediction of osteoporosis,^74,75^ detection of submandibular gland sialoliths,^73^ differentiation of Stafne’s bone cavity from mandibular radiolucent lesions^53^ on 2D or 3D radiographic images. All these models obtained high accuracies mostly with AUC values over 0.9.

### Reporting of the dental status

Charting of teeth, restorations, and present dental diseases is the first step in the routine assessment of dental patients. Any mistakes or oversights in the resulting dental records may lead to misdiagnosis and erroneous treatment decisions, such as extraction or endodontic treatment of the wrong tooth. As electronic dental health records are by now widely used in dental practice, automated charting using AI seems highly useful. Some studies reported excellent performance of CNN models for automated detection and numbering of deciduous and permanent teeth on panoramic images.^83,84^ Shaheen et al developed a CNN model on CBCT images for automated tooth segmentation and classification.^85^ The model achieved high accuracies for both segmentation and classification tasks, and has found its way into a commercially available software named Relu (Leuven, Belgium; Table 3). Fontenele et al reported that the presence of dental fillings in CBCT images negatively affected Relu’s performance on tooth segmentation.^86^ Some CNN models were developed for automated detection, segmention, and labelling of teeth, crowns, restorative fillings, root canal fillings, and dental implants.^79–82^ Commercially available systems including dentalXrai (Berlin, Germany), Denti.AI (Toronto, Canada), and Diagnocat (Tel Aviv, Israel) (Table 3) allow such charting in similar accuracy to practitioners.^78^ Moreover, CNN models were able to automatically classify various implant systems and their prosthetic status on periapical and panoramic images.^40–42^ These models achieved excellent classification accuracy and some even outperformed periodontists. Automatic implant classification models could be used to recognize and record the system of the placed implants in the dental recording systems, which can facilitate regular maintenance and future repairs.

### Treatment planning

AI has great potential to help dental practitioners with treatment planning and time-consuming tasks in the digital dental workflow. Segmentation, localization, and measurement of anatomical structures or pathologies on radiographic images as well as multimodal image registration are common manual steps required in the planning of oral and maxillofacial surgical procedures.^106^ So far, several AI applications have been proposed for automated landmark localization,^59–64^ skeletal classification,^65–67^ facial symmetry assessment,^68,107^ and decision-making on tooth retention or extraction for orthodontic treatment^108,109^ on 2D or 3D images.

Kunz et al developed a CNN model to automatically localize anatomical landmarks and measure their linear/angular parameters on cephalometric radiographs.^60^ The mean absolute differences in the linear/angular analyses were 0.44–0.64 mm/0.46–2.18° for the model and 0.35–0.88 mm/0.55–1.80° for 12 orthodontists, which demonstrates similar performance. Bulatova et al^63^ and Mahto et al^64^ tested AI driven automated cephalometric analysis software applications named Ceppro (Seoul, Korea; Table 3) and WebCeph (Seongnam, Korea; Table 3), respectively. *Ceppro* achieved mean absolute localization differences ranging from 1.3 to 8.7 mm, with no significant differences between automated and manual localization for eleven out of sixteen selected landmarks. WebCeph obtained high agreement with intraclass correlation coefficients over 0.9 between automated and manual measurements on seven out of twelve cephalometric parameters. Some deep learning models on cephalometric or CBCT images classified skeletal malocclusion for orthodontic and orthognathic treatment planning and obtained excellent accuracies over 93%.^65–67^ Lin et al developed a CNN model to assess facial symmetry before and after orthognathic surgery on CBCT images and reported high accuracy of 90%.^68^

Another group developed a CNN model for automatic detection of edentulous sites, nasal fossa, maxillary sinus, and mandibular canal, and measurement of the heights and widths of residual alveolar bone at the edentulous sites on CBCT images for dental implant treatment planning.^39^ The model’s detection accuracy was high for edentulous sites (95.3%) and moderate for the mandibular canal (72.2%) and nasal fossa/maxillary sinus (66.4%). On the sites of maxillary premolars/molars and mandibular premolars, the automated bone height measurements were similar to the manual measurements (*i.e.,* ground truth). The automated bone height measurements on the sites of maxillary/mandibular anterior teeth and mandibular molars as well as the automated bone width measurements on all tooth sites were significantly different from the manual measurements, with median measurement deviations of 1.7–11.3 mm. The significant differences between automated and manual measurements might be due to the incorrect localization of the measuring points.

Assessment of the difficulty of planned third molar surgery is also a field of increased interest in AI research. Yoo et al developed a CNN model on panoramic images to classify the difficulty of third molar removal according to several parameters, such as the depth and angulation of the molar.^47^ CNN models were also developed to classify the positional relationship between lower third molars and the mandibular canal on panoramic and CBCT images.^44–46^ Choi et al developed a CNN model to determine whether lower third molars are truly in contact with or positioned buccally/lingually to the mandibular canal when they are shown as overlapped on panoramic images (CBCT readings served as ground truth), and to classify the non-contact molars as being buccally or lingually positioned.^46^ The model obtained accuracies of 72% for determining the true contact position and 81% for classifying the bucco-lingual position, outperforming six oral-maxillofacial specialists. Kim et al developed a CNN model on panoramic images to predict paresthesia due to damage of the inferior alveolar nerve during lower third molar removal, and reported high prediction accuracy with an AUC of 0.92.^48^ Apart from third molars, Vollmer et al attempted to develop CNN models on panoramic images to predict oroantral communication after tooth extraction.^58^ The prediction accuracy of the best model was similar to that of four oral-maxillofacial experts.

Multimodal image registration is a critical step in digital dental workflows where 3D images acquired from different imaging modalities, including CT, CBCT, MRI, intraoral, facial, and model scanning, are superimposed into the same coordinate frame to create a virtual augmented patient model. This is useful for treatment planning for dental implant placement, joint, salivary gland, orthognathic, and reconstructive surgeries.^110^ Multimodal image registration can be performed manually by aligning anatomical landmarks or semi-automatically by using the surface-based or fiducial marker registration approach. Although the semi-automatic approach is less time-consuming than the manual approach, its registration accuracy is affected by the quality of the acquired images, the presence of image artifacts, the deformation of the optical surface, and the distribution of the employed fiducial markers. Therefore, manual corrections are frequently required after semi-automatic image registration. In order to improve the efficiency and accuracy of multimodal image registration, a range of studies developed AI models to automatically register CBCTs with intraoral scans,^92^ optical dental model scans,^93^ or MRIs.^94^ Compared with conventional approaches, these models allow more accurate automated image registration in a significantly shorter time.

### Image quality improvement

Due to the growing use of CBCT in daily dental practice for diagnosis and treatment planning, concerns have been raised regarding the increased risk of radiation-induced stochastic effects, particularly on radiosensitive organs such as salivary glands, thyroid glands, and eye lenses. While several low-dose CBCT protocols have been suggested and applied in practice, the perceived inferior image quality of low-dose scans may hamper their use, with clinicians nevertheless employing standard- or even high-dose scanning mode for certain imaging tasks.^111^ In addition, the presence of severe artifacts in CBCT images has been reported as one of the common reasons for re-exposure.^112^ Patient motion during scanning and metallic dental restorations are the main sources for the occurrence of movement or metal artifacts in CBCT images.

Several researchers therefore developed AI applications to correct blurred panoramic images and reduce noise and metal artifacts in CT and CBCT images.^87–90^ CNN-based auto-positioning can reduce blurring occurred due to positioning errors in panoramic image by reconstructing the image with the corrected curvature.^87^ Hu et al^91^ and Park et al^89^ developed image denoizing tools using deep learning to remove noise from low-dose CT or CBCT for improving the image quality to be equivalent to high-dose scans. Hatvani et al developed a CNN-based method to enhance the resolution of teeth on cross-sectional CBCT images, which allows better visualization of the anatomical structure of teeth.^90^ CNN-based methods were also proposed to reduce metal artifacts in CT or CBCT images.^88,113,114^ These methods identify and segment the areas of metal artifacts in the original images and then merge the original and corrected images to suppress the artifacts.

## The future of AI in dentomaxillofacial imaging

Current applications of AI in dentomaxillofacial imaging mainly focus on improving diagnostic accuracy and easing the diagnostic and/or planning workflow. More and more AI applications were reported to be able to perform similarly to or even outperformed dentists (Table 4), oftentimes lifting general dental practitioners to levels equivalent to specialists. Notably, existing AI models are limited in their scope, mainly aiming to detect, segment, or classify anatomical structures or common pathologies. Rare variations or diseases have so far seldom been the focus. Given the difficulty practitioners have for diagnosing rare variations or diseases, AI models developed for such specific tasks could be truly clinically significant. With technical advances, the availability of larger data pools (including the pooling of different datasets and the uptake of federated learning,^115^ and the increased usage of alternative labeling and training pathways in dentistry (such as weakly or self-supervised learning,^116^ these difficult diagnostic tasks are expected to be tackled.

**Table 4.** Performance of the developed AI models in comparison to specialists/general practitioners

Author (Year)	Application	Imaging modality	AI software/ deep learning model	Test dataset	Performance of the developed software/model versus human		Main findings
					AI	Human Mean (range)	
***Dental caries***							
Srivastava et al. (2017)^19^	Detection of dental caries	Bitewing radiography	CNN	500 images from nearly 100 clinics across USA	SEN = 0.81 PPV = 0.62 F1 = 0.7	*three dentists* SEN = 0.42 (0.34–0.48) PPV = 0.78 (0.63–0.89) F1 = 0.53 (0.5–0.56)	The model achieved significantly higher F1-score and sensitivity for detecting caries than three dentists.
Cantu et al. (2020)^20^	Detection of initial (enamel) and advanced (dentin) proximal caries	Bitewing radiography	CNN (U-Net)	141 bitewings from the dental clinic at Charité - Universitätsmedizin Berlin	*All caries* ACC = 0.80 SEN = 0.75 SPE = 0.83 PPV = 0.70 NPV = 0.86 F1 = 0.73 MCC = 0.57 *Initial caries* SEN > 0.7 *Advanced caries* SEN > 0.7	*seven experienced dentists* *All caries* ACC = 0.71 SEN = 0.36 (0.19–0.65) SPE = 0.91 (0.69–0.98) PPV = 0.75 (0.41–0.88) NPV = 0.72 (0.68–0.82) F1 = 0.41 (0.26–0.63) MCC = 0.35 (0.14–0.51) *Initial caries* SEN <0.25 *Advanced caries* SEN = 0.40–0.75	The model achieved higher overall accuracy than seven dentists. The seven dentists were far less sensitive, but slightly more specific than the model. For initial caries, the risk of under detection by dentists was very high while the model showed robust sensitivity regardless of the lesion depth.
Mertens et al. (2021)^99^	Detection of proximal enamel, early dentin, and advanced dentin caries	Bitewing radiography	*dentalXr.ai Pro* software	20 bitewings from the dental clinic at Charité - Universitätsmedizin Berlin	*ten images evaluated by 22 dentists with the aid of dentalXr.ai Pro* AUC = 0.89 ACC = 0.94 SEN = 0.81 SPE = 0.97 PPV = 0.82 NPV = 0.97 F1 = 0.81	*ten images evaluated by 22 dentists without the aid of dentalXr.ai Pro* AUC = 0.85 ACC = 0.93 SEN = 0.72 SPE = 0.97 PPV = 0.80 NPV = 0.95 F1 = 0.76	*dentalXr.ai Pro* software can significantly increase dentists’ sensitivity for detecting enamel caries.
Devlin et al. (2021)^21^	Detection of proximal enamel caries	Bitewing radiography	*AssistDent* software	24 images from one dental hospital and nine general dental practice sites in UK	*24 images evaluated by 12 dentists with the aid of AssistDent* SEN = 0.76 SPE = 0.85	*24 images evaluated by 11 dentists without the aid of AssistDent* SEN = 0.44 SPE = 0.96	*AssistDent* software can significantly increase dentists’ sensitivity for detecting proximal enamel caries in enamel
***Endodontic evaluation***							
Hamdan et al. (2022)^101^	Detection of apical radiolucencies	Periapical radiography	*Denti.AI* software	68 images from one dental center	*six operative dentistry residents, one general dentist and one endodontist with the aid of Denti.AI* AUC = 0.89 SEN = 0.93 SPE = 0.73	*six operative dentistry residents, one general dentist and one endodontist without the aid of Denti.AI* AUC = 0.82 SEN = 0.94 SPE = 0.60	*Denti.AI* software can enhance dental practitioner’s ability to detect apical radiolucencies on periapical images.
Jeon et al. (2021)^34^	Detection of C-shaped canals in mandibular second molars	Panoramic radiography	CNN (Xception)	408 cropped images of mandibular second molars	AUC = 0.98 ACC = 0.95 SEN = 0.93 SPE = 0.97 PPV = 0.96	*OMF radiologist/endodontist* AUC = 0.87/0.89 ACC = 0.87/0.89 SEN = 0.93/0.92 SPE = 0.82/0.86 PPV = 0.84/0.86	The model outperformed the OMF radiologist and endodontist
Sherwood et al. (2021)^35^	Segmentation and classification of C-Shaped canals in mandibular second molars	CBCT	CNN (U-Net, Residual U-Net, or XceptionU-Net)	35 scans	SEN = 0.72–0.79	*one endodontist and 1 OMF radiologist* SEN = 0.97	The model performed less well than the OMF radiologist and endodontist while it may aid clinicians with the detection and classification of C-shaped canal anatomy.
Yang et al. (2022)^36^	Classification of C-shaped canals in mandibular second molars	Periapical and panoramic radiography	CNN	100 cropped images consisting of 56 mandibular second molars without C-shaped canals and 44 molars with C-shaped canals	*Periapical images (PA*) AUC = 0.95 ACC = 0.90 SEN = 0.93 SPE = 0.87 PPV = 0.90 NPV = 0.91 F1 = 0.91 *Panoramic images (Pano*) AUC = 0.93 ACC = 0.85 SEN = 0.72 SPE = 0.93 PPV = 0.87 NPV = 0.84 F1 = 0.79	*one specialist* AUC = 0.95 (PA); 0.96 (Pano) ACC = 0.95 (PA); 0.96 (Pano) SEN = 0.95 (PA); 0.97 (Pano) SPE = 0.94 (PA); 0.95 (Pano) PPV = 0.94 (PA); 0.95 (Pano) NPV = 0.95 (PA); 0.97 (Pano) F1 = 0.94 (PA); 0.96 (Pano) *one general dentist* AUC = 0.89 (PA); 0.91 (Pano) ACC = 0.89 (PA); 0.91 (Pano) SEN = 0.91 (PA); 0.93 (Pano) SPE = 0.87 (PA); 0.89 (Pano) PPV = 0.86 (PA); 0.89 (Pano) NPV = 0.92 (PA); 0.93 (Pano) F1 = 0.89 (PA); 0.91 (Pano)	The model’s diagnostic performance using only the root portion of the tooth was similar to the specialist and superior to the general dentist. Both the specialist and general dentist showed better diagnostic performance when reading panoramic radiographs compared with periapical images.
***Periodontal evaluation***							
Kim et al. (2019)^22^	Detection of periodontal bone loss	Panoramic radiography	Deep neural transfer network	800 images from Korea University of Anam Hospital	AUC = 0.95 SEN = 0.77 SPE = 0.95 PPV = 0.73 NPV = 0.96 F1 = 0.75	*five dentists* AUC = 0.85 (0.84–0.87) SEN = 0.78 (0.74–0.80) SPE = 0.92 (0.91–0.93) PPV = 0.62 (0.59–0.65) NPV = 0.96 (0.95–0.97) F1 = 0.69 (0.68–0.70)	The model outperformed five dentists in detecting periodontal bone loss.
Krois et al. (2019)^23^	Detection of periodontal bone loss	Panoramic radiography	CNN	353 cropped images of individual tooth	AUC = 0.89 ACC = 0.81 SEN = 0.81 SPE = 0.81 PPV = 0.76 NPV = 0.85 F1 = 0.78	*six dentists* AUC = 0.77 ACC = 0.76 SEN = 0.92 SPE = 0.63 PPV = 0.68 NPV = 0.90 F1 = 0.78	The model outperformed six dentists in detecting periodontal bone loss.
***Dental implants***							
Liu et al. (2022)^37^	Detection of peri-implant bone loss	Periapical radiography	Faster R-CNN	150 images of bone level dental implants placed in patients	SEN = 0.67 SPE = 0.87 PPV = 0.81	*two dentists* SEN = 0.62–0.93 SPE = 0.64–0.77 PPV = 0.69–0.70	The model performed similarly to two dentists, but inferior to one experienced dentist (ground truth)
Lee et al. (2020)^41^	Classification of six dental implant systems	Periapical and panoramic radiography	18-layer deep CNN	2,396 cropped images of individual dental implant placed in patients from three centers including Daejeon Dental Hospital, Wonkwang University; Ilsan Hospital, National Health Insurance Service; and Mokdong Hospital, Ewha Womans University	AUC = 0.90–0.98 SEN = 0.83–0.97 SPE = 0.83–0.98	*six board-certified periodontists* AUC = 0.50–0.97 SEN = 0.78–0.97 SPE = 0.39–0.99 *eight periodontology residents* AUC = 0.50–0.92 SEN = 0.10–0.95 SPE = 0.38–0.99 *eleven residents not specialized in periodontology* AUC = 0.54–0.92 SEN = 0.49–0.89 SPE = 0.39–0.96	The model outperformed most of the participating periodontists, periodontal residents, and residents not specialized in periodontology.
***Cystic, nodal, and tumor lesions***							
Poedjiastoeti et al. (2018)^50^	Detection of ameloblastomas and keratocysts	Panoramic radiography	CNN (VGG-16)	100 images from 50 patients with ameloblastomas and 50 patients with keratocysts	ACC = 0.83 SEN = 0.82 SPE = 0.83 Diagnostic time: 38 s	*5 OMF surgeons* ACC = 0.83 SEN = 0.81 SPE = 0.83 Diagnostic time: 23 mins	The model’s performance was on par with five OMF surgeons.
Endres et al. (2020)^49^	Detection and segmentation of infection, granuloma, cysts, and tumors in the jaws	Panoramic radiography	CNN (U-Net)	102 images from the Department of Oral and Maxillofacial Surgery, Charité, Berlin	SEN = 0.51 PPV = 0.67	*24OMF surgeons* SEN = 0.51 (0.26–0.76) PPV = 0.69 (0.42–0.93)	The model outperformed 14 of 24 OMF surgeons
Ariji et al. (2022)^77^	Identification of metastatic cervical lymph nodes	Contrast-enhanced CT	CNN (U-Net)	72 image slices of 24 metastatic and 68 non-metastatic lymph nodes from 59 OSCC patients	AUC = 0.95 ACC = 0.96 SEN = 0.98 SPE = 0.95	*two radiologists* AUC = 0.90 ACC = 0.89 SEN = 0.94 SPE = 0.86	The model outperformed two radiologists in identifying metastasis with a short time period of 7 sec.
***Others***							
Kunz et al. (2020)^60^	Localization of cephalometric landmarks	Cephalometric radiography	CNN	50 images from a private orthodontic dental practice	Mean absolute differences between AI and gold standard ranging 0.46–2.18° for angular and 0.44–0.64 mm for linear analyses	Mean absolute differences between 12 orthodontists and gold standard ranging 0.55–1.80° for angular and 0.35–0.88 mm for linear analyses	The model’s performance reached the level equivalent to that of experienced orthodontists.
Ezhov et al. (2021)^78^	Segmentation of teeth and jaws, numbering of teeth, detection of caries, periapical lesions, and periodontitis	CBCT	*Diagnocat* software	30 scans selected from 1,135 scans acquired from 17 scanners	Cross-condition SEN = 0.92 SPE = 0.99	*4 OMF radiologists* Cross-condition SEN = 0.93–0.94 SPE = 0.99–1.00	*Diagnocat*‘s performance was on par with four radiologists
Choi et al. (2022)^46^	Determination and classification of positional relationships between lower third molars and the mandibular canal	Panoramic radiography	CNN (ResNet-50)	Cropped images of lower third molars with their roots overlapping the mandibular canal from 25% of 571 panoramic images	Determination of the true contact position ACC = 0.72 SEN = 0.86 SPE = 0.55 Classification of the bucco-lingual position ACC = 0.81 SEN = 0.87 SPE = 0.75	*6 OMF surgeons* Determination of the true contact position ACC = 0.53–0.70 SEN = 0.25–0.88 SPE = 0.17–0.92 Classification of the bucco-lingual position ACC = 0.32–0.52 SEN = 0.40–1.0 SPE = 0–0.56	The model outperformed six OMFS specialists with much higher accuracy for determining the true contact position and classifying the bucco-lingual position between lower third molars and the mandibular canal.
Vollmer et al. (2022)^58^	Prediction of oroantral communication after tooth extraction	Panoramic radiography	CNN (VGG16, InceptionV3, MobileNetV2, EfficientNet, or ResNet50)	60 images from patients with or without postoperative OAC	*The highest performance* (*MobileNetV2*) AUC = 0.67 ACC = 0.74 SEN = 0.43 PPV = 0.75 F1 = 0.55	*4 OMF experts* AUC = 0.55–0.71 SEN = 0.14–0.60	Although the MobileNetV2 model and one expert reached AUCs of nearly 0.7, the overall accuracy for predicting oroantral communication after tooth extraction from panoramic images was not sufficiently reliable.
Murata et al. (2019)^55^	Diagnosis of maxillary sinusitis	Panoramic radiography	CNN (AlexNet)	120 images consisting of 60 healthy and 60 inflamed sinuses	ACC = 0.88 SEN = 0.87 SPE = 0.88 PPV = 0.88 NPV = 0.87	*Radiologists/dental residents* ACC = 0.90/0.77 SEN = 0.90/0.78 SPE = 0.89/0.75 PPV = 0.89/0.76 NPV = 0.90/0.78	The model performed similarly to two OMF radiologists and outperformed two dental residents.
Jung et al. (2021)^69^	Diagnosis of temporomandibular joint osteoarthritis	Panoramic radiography	CNNs (ResNet-152 or EfficientNet-B7)	20% of 858 images from 395 patients with normal TMJs and 463 with TMJ osteoarthritis	*ResNet/EfficientNet* AUC = 0.94/0.95 ACC = 0.88/0.88 SEN = 0.95/0.86 SPE = 0.80/0.91	*Specialists/general dentists* ACC = 0.88/0.67 SEN = 0.86/0.69 SPE = 0.91/0.65	The model outperformed three general dentists and three specialists in the diagnosis of TMJ osteoarthritis
Kise et al. (2019)^76^	Diagnosis of Sjögren’s syndrome	CT	CNN (AlexNet)	100 CT slices from 5 patients diagnosed with Sjögren’s syndrome and five individuals without any parotid gland abnormalities	ACC = 0.96 SEN = 1.0 SPE = 0.92	*Experienced*/*inexperienced radiologists* ACC = 0.98/0.84 SEN = 0.99/0.78 SPE = 0.97/0.89	The model performed similarly to three experienced OMF radiologists and outperformed three inexperienced OMF radiologists.

ACC, accuracy; AI, artificial intelligence; AUC, area under the ROC curve; CBCT, cone-beam computed tomography; CT, computed tomography; CNN, convolutional neural network; DSC, Dice similarity coefficient; F1, F1-score; MCC, Matthew’s correlation coefficient; NPV, negative predictive value; OAC, Oroantral communication; OMF, oral and maxillofacial; PA, periapical images; Pano, panoramic images; PPV, positive predictive value (Precision); SEN, sensitivity (Recall); SPE, specificity; TMJ, temporomandibular joint;

In addition, it has been expected that AI at some point could “see” more on a certain image type than the human eye could. To do so, training of the AI on more sensitive sensor data as ground truth than the one later used during inference would be one option. Currently, this is not the case because the ground truth relies on the same sensor data and oftentimes involves human activity. The only way to achieve somewhat “superhuman” performance is by involving a larger number of practitioners to at least overcome the limitations of single dentists.^14^

Gradually, more and more AI models proposed have been tested by completely external image data acquired from different dental centers (Table 5) as suggested by the Artificial Intelligence in Dental Research guideline.^117^ Few models were able to achieve similar performance while most showed inferior performance on external images. Some studies have reported low cross-center generalizability of their models.^70,100^ It was noted that adding external images acquired from one dental center in the training dataset could increase the model’s performance on the images from that external center but would decrease its performance on the images from the original center. These findings indicate that although cross-center training could improve the generalizability of AI models, the proportion of the images from different centers in the training dataset is also an influencing factor associated with the trained models’ performance. Therefore, future studies should focus not only on internal testing but also on external testing. If external testing shows unfavorable outcomes, cross-center training should be considered to increase the model’s generalizability.

**Table 5.** Performance of AI models on internal and external test datasets

Author (Year)	Application	Imaging modality	AI software/ deep learning model	Internal testing		External testing		Main findings
				Test dataset	Performance	Test dataset	Performance	
Orhan et al. (2020)^31^	Detection and measurement of apical lesions	CBCT	*Diagnocat* software	N/A	N/A	109 scans from Eskisehir Osmangazi University, Faculty of Dentistry	SPE = 0.89 PPV = 0.95 F1 = 0.93	*Diagnocat* achieved high performance on external validation with no significant differences in the volumes measured by *Diagnocat* and by an OMF radiologist
Krois et al. (2021)^100^	Detection of apical lesions	Panoramic radiography	CNNs (U‐Net and EfficientNet-B5)	*Dataset A =* 150 images from Charité, Berlin, Germany *Dataset B =* 150 images from King George Medical University, Lucknow, India	Model trained with images from datasets A and tested on images from dataset A SEN = 0.48 SPE = 1.0 PPV = 0.64 F1 = 0.54 Model trained with images from datasets A&B and tested on images from dataset A SEN = 0.48 SPE = 1.0 PPV = 0.57 F1 = 0.51 Model trained with images from datasets A&B and tested on images from dataset B SEN = 0.40 SPE = 1.0 PPV = 0.54 F1 = 0.46	*Dataset B =* 150 images from King George Medical University, Lucknow, India	Model trained with images from datasets A and tested on images from dataset B SEN = 0.22 SPE = 1.0 PPV = 0.63 F1 = 0.327	The model trained with images acquired from one hospital achieved lower performance (especially lower sensitivity) when tested on images acquired from another hospital.
Zadroz ˙ny et al. (2022)^79^	Multitasking including identification of missing tooth, caries, filling, prosthetic restoration, endodontically treated tooth, residual root, apical lesion, and periodontal bone loss	Panoramic radiography	*Diagnocat* software	N/A	N/A	30 images from the Dental and Maxillofacial Radiology Department, Medical University of Warsaw, Poland	*Missing tooth* SEN = 0.96, SPE = 0.98 *Caries* SEN = 0.45, SPE = 0.98 *Filling* SEN = 0.83, SPE = 0.99 *Prosthesis* SPE = 0.96, SPE = 0.99 *Endo-treated tooth* SEN = 0.87, SPE = 0.99 *Residual root* SEN = 0.82, SPE = 1.00 *Apical lesion* SEN = 0.39, SPE = 0.98 *Periodontal bone loss* SEN = 0.80, SPE = 0.85	*Diagnocat* achieved high performance on external validation in identifying missing tooth, fillings, prosthesis, endodontically treated tooth, residual root, and periodontal bone loss, but low sensitivities for identifying caries and apical lesions.
Ezhov et al. (2021)^78^	Segmentation of teeth and jaws, numbering of teeth, detection of caries, periapical lesions, and periodontitis	CBCT	*Diagnocat* software	Cropped images from 562 scans taken using 19 scanners	Overall SEN = 0.92 SPE = 0.99	30 scans taken using three different scanners from three clinics	*12 dentists with/without the aid of the Diagnocat* Overall SEN = 0.85/0.77 SPE = 0.97/0.96	The overall sensitivity of 12 dentists with the aid of *Diagnocat* on external images was lower than that of *Diagnocat* on internal images.
De Angelis et al. (2022)^80^	Tooth numbering and detection of dental implants, prosthetic crowns, fillings, root remnants, and root canal treatment	Panoramic radiography	*Promaton* software	N/A	N/A	120 images from the Department of Oral and Maxillofacial Sciences of Sapienza University of Rome, Italy	Overall AUC = 0.94 SEN = 0.89 SPE = 0.98 PPV = 0.94 NPV = 0.97	*Promaton* achieved high overall performance on external validation
Nishiyama et al. (2021)^70^	Diagnosis of mandibular condyle fracture	Panoramic radiography	CNN (AlexNet)	*5-fold CV* *Dataset A =* 200 images from a university dental hospital *Dataset B =* 200 images from a general hospital	Model trained with and tested on images from dataset A/B AUC = 0.85/0.86 ACC = 0.80/0.81 SEN = 0.80/0.80 SPE = 0.79/0.82 Model trained by images from datasets A&B and tested on images from dataset A/B AUC = 0.89/0.91 ACC = 0.82/0.85 SEN = 0.83/0.85 SPE = 0.80/0.84	*Dataset A =* 200 images from a university dental hospital *Dataset B =* 200 images from a general hospital	Model trained with images from dataset A and tested on images from dataset B AUC = 0.58 ACC = 0.59 SEN = 0.60 SPE = 0.58 Model trained with images from dataset B and tested on images from dataset A AUC = 0.58 ACC = 0.60 SEN = 0.61 SPE = 0.59	The model trained with images acquired from one hospital achieved much lower diagnostic performance when tested on images acquired from another hospital.
Jung et al. (2021)^57^	Segmentation of maxillary sinus lesions	CBCT	CNN (3D nnU-Net)	20 scans from Korea University Anam Hospital	DSC (air) = 0.93 DSC (lesions) = 0.76	20 scans from Korea University Ansan Hospital	DSC (air) = 0.97 DSC (lesions) = 0.54	The model achieved similar performance on external images in segmenting the air space of the sinus but much lower performance in segmenting the sinus lesions

3D, three-dimensional; ACC, accuracy; AI, artificial intelligence; AUC, area under the ROC curve; CBCT, cone-beam computed tomography; CNN, convolutional neural network; CV, cross-validation; DSC, Dice similarity coefficient; F1, F1-score; N/A, not available; NPV, negative predictive value; OMF, oral and maxillofacial; PPV, positive predictive value (Precision); SEN, sensitivity (Recall); SPE, specificity.

The usefulness and efficacy of most proposed AI models in daily dental practice are still unclear based on current evidence. Although most studies reported that AI models could increase diagnostic ability of dental practitioners and reduce the time spent on time-consuming work in the treatment planning process, their true impact on real-world clinical practice is rarely discussed. In addition to a model’s accuracy, future studies should focus more on its impact on treatment decision, clinical and patient-reported outcomes, and cost-effectiveness, which may be more important to patients, providers, and healthcare organizers.^118^ Schwendicke et al performed the first cost-effectiveness analysis of an AI application for caries detection on bitewings.^14^ They reported that AI showed significantly higher sensitivity than dentists, which allows more early caries to be detected, facilitates non- or micro-invasive management of the detected lesions, and thus avoids costly late retreatments. The high cost-effectiveness of dental AI applications implies that integrating AI into clinical practice has the potential to reduce healthcare cost burden, revealing their economic impact on healthcare systems. Only clinically relevant AI tools that are capable of fulfilling technical requirements with promising financial potential can attract healthcare stakeholders to continuously support their development, optimization, and application in dental medicine.^119,120^ Therefore, future research should assess the clinical, technical, and financial aspects of cost-effectiveness of AI applications in dental medicine to demonstrate their true usefulness in daily practice.

Moreover, the impact of AI on patient-provider interaction should not be ignored. A more positive attitude regarding dental AI applications was observed in younger and more educated individuals than in older and less educated individuals.^121^ Compared with younger individuals, the elderly are more sceptical towards such advanced healthcare technologies. Providers need to frame the usage of AI individually to retain trust into the care process. The output of AI should be able to help patients to objectify any diagnosis and to support visual recognition of a lesion, which can improve patient-clinician communication and increase patients’ trust in any derived management.

## Conclusions and outlook

Personalized dental medicine should allow to provide the safest, most efficacious and efficient diagnostics and therapeutics tailored to individuals based on one’s biological, social, and behavioral characteristics. Based on current evidence, true personalized dental medicine is still far from being a reality as most evidence-based up-to-date clinical practice guidelines for the management of dental diseases still stratify individuals and lesions into risk groups, and thus assign identical management strategies to all individuals in a certain risk group. A wide range of AI applications, including several commercially available software options, have been developed based on diagnostic images to assist clinicians in the diagnosis and treatment planning of various dentomaxillofacial diseases, with performance similar or even superior to that of specialists. Although these dental AI applications are seen to have the potential to enable a more precise, personalized, preventive, and participatory approach for the management of dentomaxillofacial diseases, almost all of them only work on image data obtained at a certain time point in the diagnostic or treatment process without considering other data such as individual characteristics and clinical assessment. Advanced technologies with improved data analytic approaches are expected to enrich these AI applications with diverse, multimodal, large, and complex data from the individual level (*e.g.,* demographic, behavioral, and social characteristics; clinical data generated by records mining, clinical assessment, diagnostic imaging, omics technologies; and real-time consumer data from wearables and tracking devices), setting level (*e.g.,* geospatial, environmental, and provider-related data), and system level (*e.g.,* health insurance, regulatory, and legislative data), which may facilitate a deeper understanding of the interaction of these multilevel data and hopefully bring us closer to a truer form of personalized dental care for patients in the near future.^3,7^
